# Methodological heterogeneity biases physical activity metrics derived from the Actigraph GT3X in multiple sclerosis: A rapid review and comparative study

**DOI:** 10.3389/fresc.2022.989658

**Published:** 2022-11-28

**Authors:** Ashley Polhemus, Christina Haag, Chloé Sieber, Ramona Sylvester, Jan Kool, Roman Gonzenbach, Viktor von Wyl

**Affiliations:** ^1^ Epidemiology and Biostatistics and Prevention Institute, University of Zürich, Zürich, Switzerland; ^2^ Institute for Implementation Science in Health Care, University of Zürich, Zürich, Switzerland; ^3^ Research Department Physiotherapy, Rehabilitation Centre, Valens, Switzerland

**Keywords:** actigraph, physical activity, step count, methodology, multiple sclerosis

## Abstract

**Background:**

Physical activity (PA) is reduced in persons with multiple sclerosis (MS), though it is known to aid in symptom and fatigue management. Methods for measuring PA are diverse and the impact of this heterogeneity on study outcomes is unclear. We aimed to clarify this impact by comparing common methods for deriving PA metrics in MS populations.

**Methods:**

First, a rapid review of existing literature identified methods for calculating PA in studies which used the Actigraph GT3X in populations with MS. We then compared methods in a prospective study on 42 persons with MS [EDSS 4.5 (3.5–6)] during a voluntary course of inpatient neurorehabilitation. Mixed-effects linear regression identified methodological factors which influenced PA measurements. Non-parametric hypothesis tests, correlations, and agreement statistics assessed overall and pairwise differences between methods.

**Results:**

In the rapid review, searches identified 421 unique records. Sixty-nine records representing 51 eligible studies exhibited substantial heterogeneity in methodology and reporting practices. In a subsequent comparative study, multiple methods for deriving six PA metrics (step count, activity counts, total time in PA, sedentary time, time in light PA, time in moderate to vigorous PA), were identified and directly compared. All metrics were sensitive to methodological factors such as the selected preprocessing filter, data source (vertical vs. vector magnitude counts), and cutpoint. Additionally, sedentary time was sensitive to wear time definitions. Pairwise correlation and agreement between methods varied from weak (minimum correlation: 0.15, minimum agreement: 0.03) to perfect (maximum correlation: 1.00, maximum agreement: 1.00). Methodological factors biased both point estimates of PA and correlations between PA and clinical assessments.

**Conclusions:**

Methodological heterogeneity of existing literature is high, and this heterogeneity may confound studies which use the Actigraph GT3X. Step counts were highly sensitive to the filter used to process raw accelerometer data. Sedentary time was particularly sensitive to methodology, and we recommend using total time in PA instead. Several, though not all, methods for deriving light PA and moderate to vigorous PA yielded nearly identical results. PA metrics based on vertical axis counts tended to outperform those based on vector magnitude counts. Additional research is needed to establish the relative validity of existing methods.

## 1 Introduction

Multiple sclerosis (MS) is a neurodegenerative autoimmune disease which affects individuals' physical and cognitive function, motor control, and energy levels. Physical activity (PA) is known to aid in symptom management and improve quality of life in individuals with MS (1, 2). However, PA is reduced in this population compared to healthy controls (3, 4). Interventions which can increase PA in individuals with MS, for example through rehabilitation (5), exercise training (2), or behavior modification coaching (6), are therefore a topic of public health and research interest (6). However, if we are to understand PA behavior in MS populations and to evaluate the efficacy of these interventions, we require PA outcomes measures which are valid, responsive, and feasible to implement.

Many outcome measures have been used to measure PA in populations with MS (7, 8), though the optimal PA metrics for MS populations are not yet clear (6). Patient reported measures, such as the International Physical Activity Questionnaire (IPAQ) (9) or Godin's Leisure Time Exercise Questionnaire (GLTEQ) (10), are inexpensive and validated in MS, but are burdensome to complete regularly, subject to recall bias, and insensitive to short bouts of light or lifestyle PA (11–14). Further, these measures assess participants' perceptions of their own PA behavior, rather than the PA they objectively completed. Alternatively, objective metrics derived from wearable sensors can quantify PA unobtrusively during daily life with minimal input from the wearer. The Actigraph GT3X is the most common sensor used for PA research with persons with MS (PwMS) (7). Unlike most other PA trackers, the Actigraph is capable of deriving step counts, PA intensity, and other metrics in a flexible manner, allowing researchers substantial freedom in the way they process data and calculate PA.

While this flexibility allows the Actigraph to be used in many settings and patient populations, it also contributes to substantial methodological heterogeneity in the PA literature. In a systematic review, Migueles et al. mapped the sensor settings and data processing methods used to derive PA metrics from the Actigraph for healthy populations (15). These differences included various sensor placements, data filtering methods, wear time definitions, and cutpoints (i.e., thresholds which differentiate sedentary behavior, light PA [LPA], and moderate to vigorous PA [MVPA]). These methodological factors have the potential to affect the outcomes of Actigraph-derived PA metrics at various points throughout study conduct and data analysis (Table 1). In the MS literature, the extent of this methodological heterogeneity and its practical implications, both for study design and the interpretation of existing literature, are currently unclear.

**Table 1:** Methodological factors which can affect PA metrics derived from the Actigraph GT3X during device initialization, study conduct, and data processing.

Implementation phase	Methodological factors	Description
Device Initialization	Sampling frequency	The Actigraph measures acceleration a set number of times per second (i.e., the sampling frequency), generating raw data in three axes, which correspond to movement along the body's longitudinal, sagittal, and frontal axes. The sampling frequency is selected before the device is given to the participant (16).
Study Conduct	Sensor placement	The Actigraph can be worn on either hip, wrist, or ankle. The locations do not produce equivalent data, and data processing methods are generally validated with respect to a specific sensor placement (17, 18).
Data Processing		
Filter data	Filter	During processing, a bandpass filter is applied to the raw data to remove non-physiological movement artifacts. Two settings are available: a standard filter for healthy gait and the low-frequency extension (LFE) for populations with impaired gait. The LFE is designed to be more sensitive to light, slow movements (16).
Aggregate into epochs	Epoch length	Filtered data is aggregated into epochs, which can be seconds, minutes, or longer in length. The resulting data is expressed in terms of the number of activity counts or steps which occurred during each epoch. Many algorithms are designed to be used with specific epoch lengths (16).
Identify non-wear time	Wear time algorithm Inactivity period	Data is then further processed through wear time algorithms to differentiate between inactive periods when the wearer was sedentary, and those when the device was not worn. This typically consists of identifying extended periods with zero activity counts, with or without a spike tolerance (19).
Remove invalid days	Minimum daily wear time	A minimum daily wear time is typically specified to ensure that PA metrics are valid representations of the wearer's daily activity. Days which did not reach this minimum are usually considered invalid and removed from the dataset (16).
Remove invalid measurements	Minimum valid days Minimum weekdays vs. weekend days	Daily PA metrics are frequently averaged over the course of a week to account for day-to-day variability. A minimum number of wear days is typically specified to ensure measures are not confounded by this variability. Studies sometimes require that measurements do or do not contain a weekend day, as activity can differ from weekdays (20).
Calculate physical activity metrics	Data source Algorithms, cutpoints, and metric definitions	Algorithms and cutpoints (i.e., limits used to differentiate between PA intensity levels) are applied to the data to calculate PA metrics. These methods may use raw or epoch-level data, vertical axis activity counts, vector magnitude counts, step counts, or other data sources to calculate PA metrics. Further aggregation and processing are then applied to calculate outcome measures, such as average daily metrics (16). Researchers may opt to adjust PA metrics for wear time if wear time is contributing to intra- and inter-participant variability (21).

The objective of this study was to quantify the extent and the potential confounding effects of methodological heterogeneity on PA metrics derived from the Actigraph GT3X in MS populations. First, we conducted a rapid review to assess the methodological heterogeneity of the literature and to identify common data processing methods. We then directly compared these data processing methods by applying them on a single dataset, quantifying the impact of heterogeneity on six PA metrics. These analyses identified biases arising from methodological heterogeneity and suggest implications for the implementation, interpretation, and meta-analysis of PA metrics in MS populations.

## 2 Methods

### 2.1 Rapid review

The purpose of this review was to rapidly identify established data processing methods and their common derivatives for calculating PA metrics with the Actigraph GT3X in PwMS. It was modeled after the systematic review conducted by Migueles et al. (15), and adhered to rapid review conduct guidelines (22, 23). In July 2020 and again in September 2021, we searched PubMed, Embase, IEEE Xplore for peer-reviewed and gray literature published in the English language during or after 2010, reflecting the release of the Actigraph GT3X in mid-2009 (15) and its validation for MS populations in 2012 (24, 25). We supplemented this corpus with the first 100 hits from Google Scholar and with manual reference searches. All search strategies are provided in the supplementary materials. Records were eligible if they reported using the Actigraph GT3X to measure PA in a population which included adult PwMS in the English language. Any study design, including research protocols and validation studies, were eligible. Eligibility did not depend on study setting, PA metrics, diagnostic criteria, disease severity, or disease duration. Records were screened and managed in DistillerSR software.

A single reviewer screened all abstracts and full texts for eligibility, critically appraised records, and extracted data. In contrast to systematic reviews, single-screening may be used for rapid reviews (22, 23). To our knowledge, no appraisal tool exists for critically appraising technology implementation methods. Therefore, we assessed quality through two questions:

1. Were sensor-related methods sufficiently clear to be reproducible?
2. Did the study adhere to previously validated methods? If not, did they provide novel evidence of validity?

The same single reviewer extracted data from each eligible study, including a brief description of the study design, aims, and findings, characteristics of the study population, device placement, sampling frequency, filter settings, non-wear time definition, derived PA metrics, cutpoints used for activity intensity classification, and other algorithms used to calculate PA measurements. Multiple records arising from the same study were reported separately, as they frequently reported different data processing and analysis methods. The relationships between records arising from the same study are noted in the supplementary materials. Methodological heterogeneity was evaluated qualitatively through frequency analysis (i.e., number of studies which used a given method) and narrative analysis. Frequency analysis was conducted both overall and per PA metric to identify whether methodological heterogeneity was consistent across metrics. No further sensitivity analyses or risk of bias analyses were conducted.

The protocol was finalized prior to study conduct, but was not registered as systematic review repositories do not accept rapid review protocols. No amendments were made after the protocol was finalized.

Common data processing methods, or those repeatedly used in the literature, were identified based on frequency analysis and the references in included studies. For the purposes of this study, we refer to a “method” as the unique combination of methodological factors (i.e., filter, data source, cutpoint, wear time definition, etc.) that were used to calculate each point estimate of each PA “metric” (i.e., step count, time in PA, sedentary time, etc.).

### 2.2 Experimental study

#### 2.2.1 Study participants and procedures

This study is a sub-study of BarKA-MS, a cohort study exploring barriers to PA in PwMS during and after neurorehabilitation (26). We recruited a convenience sample of PwMS undergoing elective inpatient neurorehabilitation at the Kliniken Valens between January and November 2021. Participants were eligible if they (1) had a clinically confirmed diagnosis of MS, (2) were 18 years of age or older, (3) had reduced walking ability but were able to walk independently with or without an assistive device, (4) had access to WiFi and a mobile device in the rehabilitation center and at home, (5) were willing to wear study devices to measure their PA, and (6) were able to answer study questionnaires in German. The ethics committee of the canton of Zurich approved the study protocol (BASEC-no. 2020-02350) and all participants provided written informed consent.

Screening, enrollment, and baseline visits were conducted at the rehabilitation clinic. Participants then completed personalized rehabilitation programs, which lasted between two and four weeks. For the purposes of this analysis, we collected the following outcome measures at the end of participants' rehabilitation stay: Extended disability status scale (EDSS) score (27), the 10 meter gait speed test (10 mWT) (28), the 6 min walk test (6 MWT) (29), the Multiple Sclerosis Walking Scale – 12 (MSWS-12) (30), a measure of walking ability and its impact on daily activities, and the International Physical Activity Questionnaire (IPAQ) (9), a self-assessment of PA in the previous seven days which was previously validated in PwMS.

Approximately one week prior to discharge, participants were fitted with an Actigraph GT3X accelerometer (Manufacturing Technology, Inc., FL, USA), which was worn on the non-dominant hip for the last week of the rehabilitation stay. The day the device was provisioned and the day participants were discharged were not included in the analysis, leaving most participants with a potential measurement duration of five to seven days. Participants occasionally wore the devices for a longer period if their rehabilitation was unexpectedly extended. Devices were initialized in Actilife Version 6.0 at a sampling rate of 30 Hz.

We opted to use data collected during rehabilitation, rather than in the home environment, in our primary analyses because participants were expected to engage in a diverse set of structured and unstructured physical activities during their rehabilitation programs. This choice allowed us to study PA metrics on a dataset which was known to capture sedentary behavior, LPA, and MVPA, with high wear compliance. Data collected in the home exhibited lower wear compliance and fewer valid days, with additional participants not meeting minimum wear requirements. However, the this also means that the average PA metrics presented here are not necessarily representative of participants' normal behavior, and should not be interpreted as such.

#### 2.2.2 Data processing

Data were uploaded to Actilife, processed *via* two filters [the standard filter and the low frequency extension (LFE)] and aggregated into 60 s epochs. Actigraph's proprietary step detection algorithm counted steps per minute on data derived from each filter. Data were then exported for processing in R (Version 4.1.0). Individual epochs were then categorized as sedentary, LPA, or MVPA according to the methods identified in the review (see review results, Table 3). This process yielded multiple sets of data for each PA metric (Step count: 2 sets, activity counts: 4 sets, total time in PA: 12 sets, sedentary time: 12 sets, time in LPA: 18 sets, time in MVPA: 8 sets).

Though we tested the impact of wear time definitions on PA metrics, our primary definition of non-wear time was 60 min of continuous zeros. Days with at least 10 h of wear time during waking hours (6AM to 11PM) were considered valid and a minimum of two valid days was required for inclusion (31, 32). We did not require measurements to include weekend days. This definition was used to determine whether participants met minimum wear time requirements, and was the default wear time definition used in analyses which did not assess the effects of wear time definition. We then calculated wear time *via* each combination of wear time algorithms and inactive periods (*n* = 10) for each data set derived for each PA metric. Epochs during which the Actigraph was not worn were removed. This process resulted in a unique data set which represented each method, or unique combination of methodological factors (Step count: 20 sets, activity counts: 40 sets, total time in PA: 120 sets, sedentary time: 120 sets, time in LPA: 180 sets, time in MVPA: 80 sets). For each data set, each participant's epoch-level data were then aggregated into daily PA metrics by adding all epoch-level PA during valid wear time over the course of waking hours each day. Daily metrics were then averaged over all valid days to generate average PA metrics. Average PA metrics were used in all analyses.

In some cases, researchers adjust PA metrics for wear time by expressing PA either as ratio (i.e., PA per unit wear time) which can be scaled to reflect standardized daily PA metrics to account for unequal wear times across days and across participants, reducing variability at the participant and population level (21). However, if methodological heterogeneity induces bias in wear time estimates, this method has the potential to further bias PA metrics. To quantify these effects, we generated a separate dataset by dividing each daily PA metric by the wear time measured on that day according to each of the possible wear time estimates. To allow for comparability across adjusted and non-adjusted PA metrics, we assumed a standard daily wear length of 13 h and multiplied the PA ratios by this standardized day length. We chose 13 h as a “standard” day length because this was approximately the average daily wear time of the Actigraph in our study. We adopted this approach, rather than using a proportion of wear time, to enable better comparability across adjusted and unadjusted PA metrics.

#### 2.2.3 Statistical analysis

The aims of the statistical analyses were twofold: (1) to identify the methodological factors which independently influenced PA estimates and (2) to understand the potential confounding effects of methodological heterogeneity. All statistical analyses were conducted in R (Version 4.1.0) using the RStudio environment (Version 1.4.1717).

First, multivariate linear regression with participant-level random intercepts was used to identify methodological factors which independently influenced PA metrics. For each PA metric, all replicate data sets were combined and the methodological factors used to derive these sets were treated as independent variables. Data were derived from the same raw accelerometer data and therefore highly inter-dependent, making models at risk for over-fitting. Therefore model development was based on theoretical knowledge and Bayesian Information Criterion (BIC), rather than *p* values. A base model tested the effects wear time algorithm, inactivity period definitions, data source, filter, and cutpoints, as relevant for each metric (see review results, Table 3). Each base model was adjusted for the participants' MS severity, defined as mild (EDSS < 4.0), moderate (EDSS 4.0–5.5), and severe (EDSS 6.0–6.5) body function impairment, consistent with previous studies (33). Interactions between methodological factors were considered plausible, as was effect modification by MS severity. We therefore tested for interactions between model terms through a manual forward stepwise procedure. Interactions between methodological factors were tested first, followed by interactions between methodological factors and disease severity strata. Interaction terms were retained if the more complex model exhibited a lower BIC than the previous, simpler model. We refer to models containing all methodological factors and eligible interaction terms as “fully adjusted” models. When methodological factors did not contribute substantially to these models, (i.e., when coefficients were smaller than 5% of the intercept and the factor did not exhibit interactions), these terms were removed from the model in a backward stepwise manner according to BIC. When terms did not contribute substantially to the fully adjusted model, the process was repeated in a reduced dataset which excluded all combinations of methods with these variables. The resulting models are referred to as “reduced” models. Reduced models were considered the primary models for this analysis, but both sets of models are reported in the supplementary material. This conservative model development process reduced the risk of over-fitting and spurious findings related to the close relationship between replicate data sets. All *p*-values are reported, and were corrected for multiple testing through a Benjamini Hochberg procedure (34). The R package lmerTest was used to build these models. This process was repeated for PA metrics which were adjusted for wear time.

Subsequent analyses were limited to combinations of methodological factors which were included in unadjusted PA metrics' reduced models. For each PA metric, Friedman tests identified the presence of significant differences between methods. Subsequent pairwise analyses identified median differences, statistically significant differences (Wilcoxon tests), correlations (Pearson's correlation), and agreement [Lin's concordance correlation coefficient (35) (CCC)] between each pair of methods. With the exception of sedentary time estimates, the effects of wear time algorithm and inactivity period were not included in this analysis, as they did not substantially affect PA estimates (see results). All *p*-values were corrected for multiple testing through a Benjamini Hochberg procedure (34). All pairwise analyses used the R package stats, except for the CCC analyses which used the package DescTools.

To illustrate the potential confounding effects of methodological heterogeneity, we conducted several analyses which are relevant to recent MS literature and policy development. First, we explored the impact of methodological heterogeneity on the associations between PA metrics and five clinical outcome measures (IPAQ, EDSS, 10 meter gait speed, 6MWT, and MSWS-12) which are known to associate with PA. We calculated the Spearman correlation between these clinical measures and all PA metrics and methods. We then made qualitative comparisons between point estimates of the correlations across methods for each metric. In addition, several studies have investigated whether people with MS meet minimum PA recommendations (36, 37). The National MS Society recommends that ambulatory PwMS take 7,500 steps per day or engage in 150 min of MVPA per week (i.e., 30 min per day 5 times per week) (3). We therefore compared the proportion of the study population which met these guidelines according to each method for deriving step count and MVPA. The other metrics assessed here do not have established guidelines, and were not assessed in this analysis.

## 3 Results

### 3.1 Rapid review

#### 3.1.1 Assessing methodological heterogeneity in the literature

**Figure 1:**
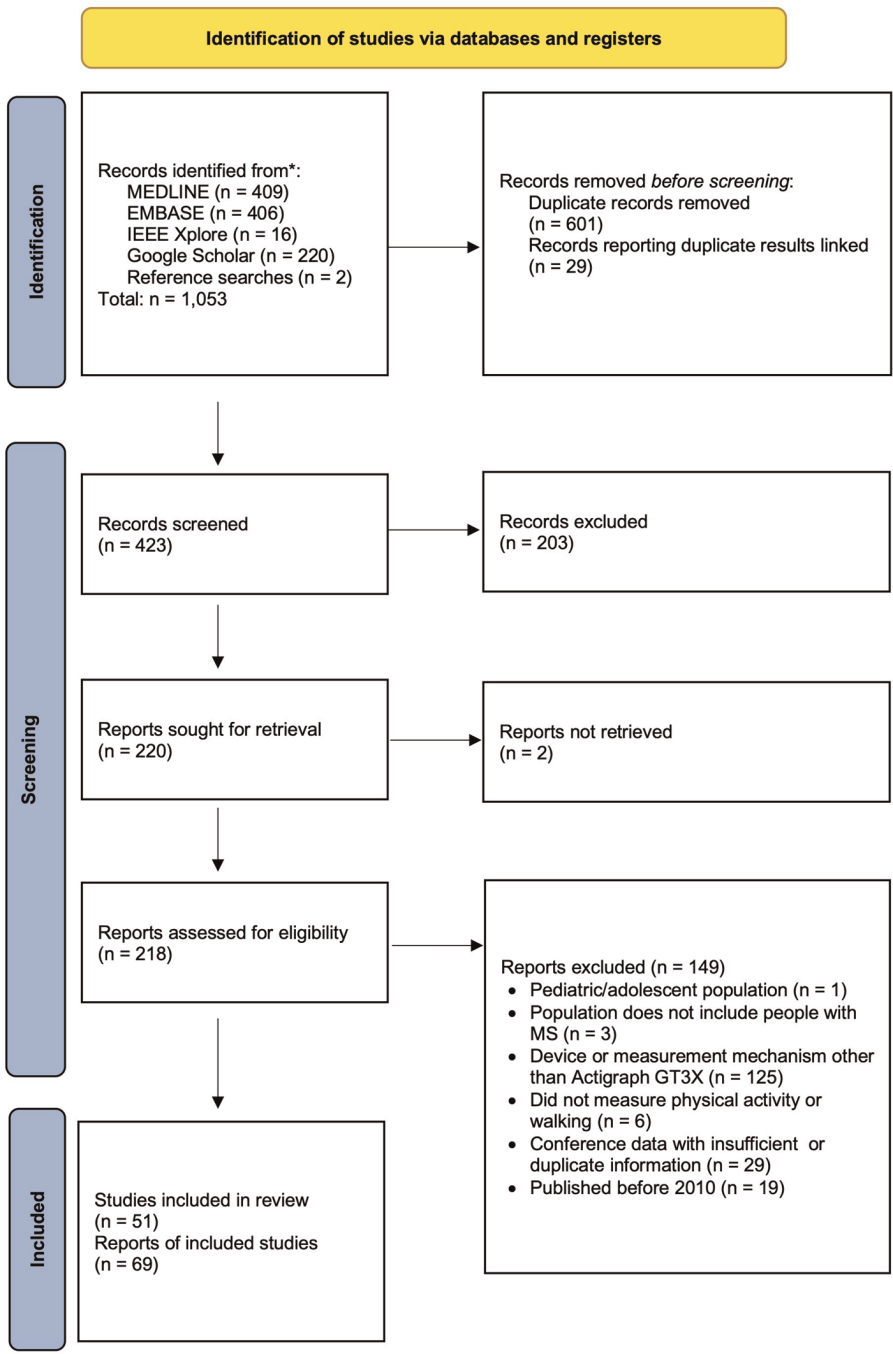
PRISMA diagram of records screened in the rapid review.

Searches identified 421 unique records, of which 216 were included in full text review. From these, 69 records from 51 unique studies were eligible (Figure 1) (20, 24, 25, 33, 38–101). Characteristics and critical appraisals of included records are reported in Supplementary Table S2, and all following results are reported in terms of the number of records which used a given method. Most records (*n* = 63) used the Actigraph with ambulatory PwMS during daily life, though two records monitored PA of wheelchair users and 12 used the devices during scripted, in-clinic walking tasks.

PA metrics included step counts (*n* = 26), activity counts (*n* = 17), sedentary behavior (*n* = 22), time spent in LPA (*n* = 22), time in MVPA (*n* = 36), total time in PA (*n* = 1), and energy expenditure (*n* = 1), though two records did not report which metrics were used. Most records reported daily PA metrics (*n* = 63), though they occasionally studied intra-day patterns (*n* = 7). The preprocessing filters, data sources, and cutpoints used to derive these PA metrics varied (Table 2). Records differed in their device placement (Hip: 43, Wrist: 7, Unspecified: 19), epoch lengths (1 s to 1 min, 26 unspecified) and sampling frequency settings (30 Hz–100 Hz, 44 unspecified). Most records' methods were not sufficiently reported to be reproducible (*n* = 64). Forty-nine explicitly followed existing methods, but 42 of these had reporting gaps. Nine studies modified established methods without further validation. Common modifications included changing the filter used to derive cutpoint-based metrics (*n* = 5) and using methods developed for hip sensor placement on wrist-derived data (*n* = 3). Three records established new methods for deriving PA metrics. A searchable and filterable table detailing methods used by each study is provided in Supplementary Table S2.

**Table 2:** Frequency table of the methods used to derive physical activity metrics identified in the rapid review.

	Step count (*n* = 26)	Activity count (*n* = 17)	Time in PA (*n* = 1)	Sedentary behavior (*n* = 22)	Time in LPA (*n* = 22)	Time in MVPA (*n* = 36)
Sensor location						
Hip	14	8	1	15	14	23
Wrist	3	5	0	2	2	6
Unspecified/Unclear	9	4	0	5	6	7
Preprocessing filter						
Standard (88)	0	0	0	0	0	1
LFE (88)	6	3	0	9	9	10
Unspecified/Unclear	20	14	1	13	13	25
Data source						
Vertical axis	–	3	0	15	15	20
Vector magnitude	–	9	0	5	5	7
Step counts	–	–	–	–	–	1
Unspecified/Unclear	–	5	1	2	2	8
Cutpoint – Sedentary behavior vs. LPA						
100 Vertical cpm (102)	–	–	0	15	15	–
150 VM cpm (102)	–	–	0	4	4	–
200 VM cpm	–	–	0	1	1	–
Unspecified/Unclear	–	–	1	2	2	
Cutpoint – Light PA vs. MVPA						
1,584 vertical cpm^a^ (24)	–	–	–	–	5	8
1,722 vertical cpm^a^ (24)	–	–	–	–	5	5
1,745 vertical cpm^a^ (23)	–	–	–	–	0	2
EDSS 0-5.5: 1,980 vertical cpm^a^ (23) EDSS 6.0+: 1,185 vertical cpm^a^	–	–	–	–	2	4
1,952 vertical cpm (103)	–	–	–	–	3	3
2,690 VM cpm (104)	–	–	–	–	5	5
EDSS 0-3.5: 99 step/min (105) EDSS 4.0-5.5: 89 steps/min EDSS 6.0+: 79 steps/min	–	–	–	–	–	1
3,644 VM cpm (69)^b^	–	–	–	–	–	2
Unspecified/Unclear	–	–	–	–	2	8
Wear time algorithm						
Continuous zeros	8	4	0	6	6	7
Troiano	2	1	0	4	4	4
Choi	1	1	0	1	0	1
Not used/Not applicable	1	0	0	0	0	3
Unspecified/Unclear	11	11	1	11	12	21
Wear time inactivity period						
30 min	0	0	0	3	2	2
60 min	10	3	0	4	5	5
90 min	1	1	0	1	0	2
180 min	0	2	0	3	3	3
Not used/Not applicable	1	0	0	0	0	3
Unspecified/Unclear	11	11	1	11	12	21

The table reflects the number of records which reported using each method. Some records used multiple methods, therefore sums may exceed the total number of records using each PA metric.

^a^ Multiple cutpoints were developed in a single study. Often, studies reference the original paper without designating which cutpoint or Actigraph settings were used.

^b^ Derived for MVPA during wheelchair propulsion. All other cutpoints were derived for ambulatory individuals.

LFE, low frequency extension, VM, vector magnitude; cpm, counts per minute; PA, Physical activity; EDSS, Expanded disability status scale; LPA, Light physical activity; MVPA, Moderate to vigorous physical activity.

Studies also varied in their wear time definitions. Most of the 63 records which described real-world PA (as opposed to in-clinic tasks) did not fully report their wear time definitions (*n* = 35). Of those that did, studies defined inactive periods of 30 min (*n* = 5), 60 min (*n* = 17), 90 min (*n* = 3), or 180 min (*n* = 3) as non-wear time. Inactive periods were either defined as periods of continuous zeros (*n* = 18), or calculated through two wear time algorithms: the Troiano algorithm (*n* = 8) or the Choi algorithm (*n* = 2). The Troiano algorithm allows for 2-minute spikes before an inactive period is broken, while the Choi algorithm allows for 2 min spikes but also requires 30 min of continuous zeros before and after spikes to qualify as a non-wear period (19, 107). One additional study defined valid days as those with 300 or more steps, and did not differentiate between wear and non-wear time. Minimum daily wear time ranged from 10 to 16 h (20 unspecified) and several studies (*n* = 16) considered days invalid if they contained any non-wear periods. Studies also differed in the minimum number of valid days required to consider a participant's measurement valid (1 to 6 days, 31 unspecified), and whether they differentiated between weekdays and weekend days in their assessments of measurement validity (57 unspecified). This information is provided in greater detail in Supplementary Table S2.

#### 3.1.2 Method selection

Based on review findings, we selected established methods and their common derivatives to quantify the potential effect of methodological heterogeneity on six PA metrics: step count, activity counts, total time in PA, sedentary time, time in light PA, and time in MVPA (24, 25, 33, 64, 85, 104–106). Due to the characteristics of our study population, we limited this comparison to methods which were designed for ambulatory individuals, rather than those who regularly use wheelchairs. In addition, we limited our analysis to methods designed for a hip sensor location as this was both the most commonly-used location and the location for which most methods were designed.

In particular, we assessed the effects of preprocessing filter (Standard filter or LFE), data source (Vertical axis counts, VM counts, or in some cases step count), three cutpoints to differentiate between sedentary and non-sedentary behavior (100, 150, and 200 cpm), and seven methods to differentiate between MVPA and non-MVPA (Tables 3, 4). We also assessed the effect of wear-time definitions on these metrics, considering three wear time algorithms [continuous zeros (Zeros), Troiano, Choi] (19, 107) and four inactivity periods (30, 60, 90, and 180 min) which were commonly used by included studies. Other methodological factors, such as the definition and number of valid days required for a valid reading (Table 1), have been addressed previously and were not studied here (31, 32).

**Table 3:** Methodological factors with the potential to affect six PA metrics derived from the Actigraph GT3X.

	Step count	Activity counts	Time in PA	Sedentary time	Time in LPA	Time in MVPA
Filter • Standard • LFE	X	X	X	X	X^a^	X^a^
Data source • Vertical axis • Vector magnitude • Steps	X	X	X	X	X^a^	X^a^
Cutpoint – Sedentary • 100 cpm • 150 cpm • 200 cpm			X	X	X	
Cutpoint – MVPA • Sandroff-1584 • Sandroff-1722 • Sandroff-1745 • Sandroff-Severity • Freedson • Sasaki • Agiovlasitis (Standard filter)^b^ • Agiovlasitis (LFE)^b^					X	X
Wear time algorithm • Zeros • Choi • Troiano	X	X	X	X	X	X
Inactivity period • 30 min • 60 min • 90 min • 180 min	X	X	X	X	X	X

^a^ MVPA cutpoints are designed to be used with specific filters and data sources, therefore the independent effect of these factors on PA metrics which utilize MVPA cutpoints were not directly assessed. Rather, these factors are implicit to the employed MVPA cutpoints as described in Table 4.

^b^ Step count based methods are not available to differentiate between sedentary and non-sedentary minutes, therefore the Agiovlasitis cutpoints cannot be used to estimate LPA.

LFE, low frequency extension; cpm, counts per minute; PA, Physical activity; LPA, light physical activity; MVPA, moderate to vigorous physical activity.

**Table 4:** Methods for deriving moderate to vigorous activity metrics in ambulatory people with multiple sclerosis.

Metric & Method	Filter	Data source	Cutpoint	Original target population
Sandroff-1,584 (24)	Standard	Vertical axis	1584 cpm	Individuals with MS
Sandroff-1,722 (24)	LFE	Vertical axis	1722 cpm	Individuals with MS
Sandroff-1,745 (23)	LFE	Vertical axis	1745 cpm	Individuals with MS
Sandroff-Severity (23)	LFE	Vertical axis	EDSS 0-5.5: 1980 cpm	Individuals with MS
			EDSS 6.0+: 1185 cpm	
Freedson (103)	LFE	Vertical axis	1952 cpm	Healthy adults
Sasaki (102, 104)	Standard	VM	2690 cpm	Healthy adults
Agiovlasitis (105)	Standard or LFE	Steps	EDSS 0-3.5: 99 steps/min	Individuals with MS
			EDSS 4.0-5.5: 89 steps/min	
			EDSS 6.0+: 79 steps/min	

LFE, low frequency extension; VM, vector magnitude; cpm, counts per minute; PA, Physical activity; EDSS, Expanded disability status scale.

### 3.2 Experimental study

#### 3.2.1 Participant characteristics

Of the 47 enrolled participants, 2 left rehabilitation early and were excluded from the study. One additional Actigraph data file was corrupted and two participants did not meet minimum wear time requirements, leaving 42 datasets for analysis. Participant characteristics are presented in Table 5. According to our primary non-wear time definition (60 min of continuous zeros), participants wore the Actigraph devices for a median of 13.7 h on 5.8 days. However, wear time estimates varied according to wear time algorithm and inactivity period length (*p* < 0.001), and estimates ranged from an average of 4.4 valid days with 12.1 h of wear time per day (Troiano algorithm, 30 min of inactivity) to 5.9 days with 14.2 h of wear time per day (180 min of continuous zeros).

**Table 5:** Participant characteristics.

Participants	42
Age	46 [40–51]
Sex (Females)	27 (64.3)
EDSS score	4.5 [3.5–6]
Mild	13 (31)
Moderate	17 (40)
Severe	12 (29)
MSWS-12 score	52 [27–67]
6MWT (m) (*n* = 33)	345 [242–428]
No walking aid	22 (67)
2 Sticks	9 (27)
Rollator	1 (3)
Other	1 (3)
10 mWT (s) (*n* = 40)	9 [7–13]
No walking aid	26 (65)
2 Sticks	8 (20)
Rollator	2 (5)
Other	4 (10)
10 m Gait speed (*n* = 41)	
>1.0 m/s	25 (61)
0.6–0.9 m/s	11 (27)
<0.6 m/s	5 (12)

Characteristics reported as *N* (%) or Median [Q2–Q4].

#### 3.2.2 Wear time

According to our primary non-wear time definition (60 min of continuous zeros), participants wore the Actigraph devices for a median of 13.7 h on 5.8 days. However, linear regression showed that wear time estimates were affected by choice of filter (Standard vs. LFE), wear time algorithm (Zeros, Choi, Troiano), and inactivity period (30, 60, 90, or 180 min). Filter and algorithm terms exhibited interactions with inactivity period. Specifically, the wear time estimates substantially decreased when a 30 min inactivity period was used with the Troiano algorithm, though use of the LFE decreased this effect. Model coefficients are provided in Supplementary Table S3 for wear time and all PA metrics. Estimates ranged from an average of 4.4 valid days with 12.1 h of wear time per day (Troiano algorithm, 30 min of inactivity) to 5.9 days with 14.2 h of wear time per day (180 min of continuous zeros).

#### 3.2.3 Step count

Linear mixed effects modeling showed that choice of filter (Standard vs. LFE) affected raw daily step count estimates, whereas wear time algorithm and inactivity period did not. MS severity acted as an effect modifier with respect to the impact of the filter; the LFE disproportionately increased step counts in severe MS compared to mild and moderate MS (Supplementary Table S3). When step counts were adjusted for wear time, they became independently affected by wear time algorithm and inactivity period, with the Troiano algorithm and a 30 min inactivity period imparting the largest biases.

**Figure 2:**
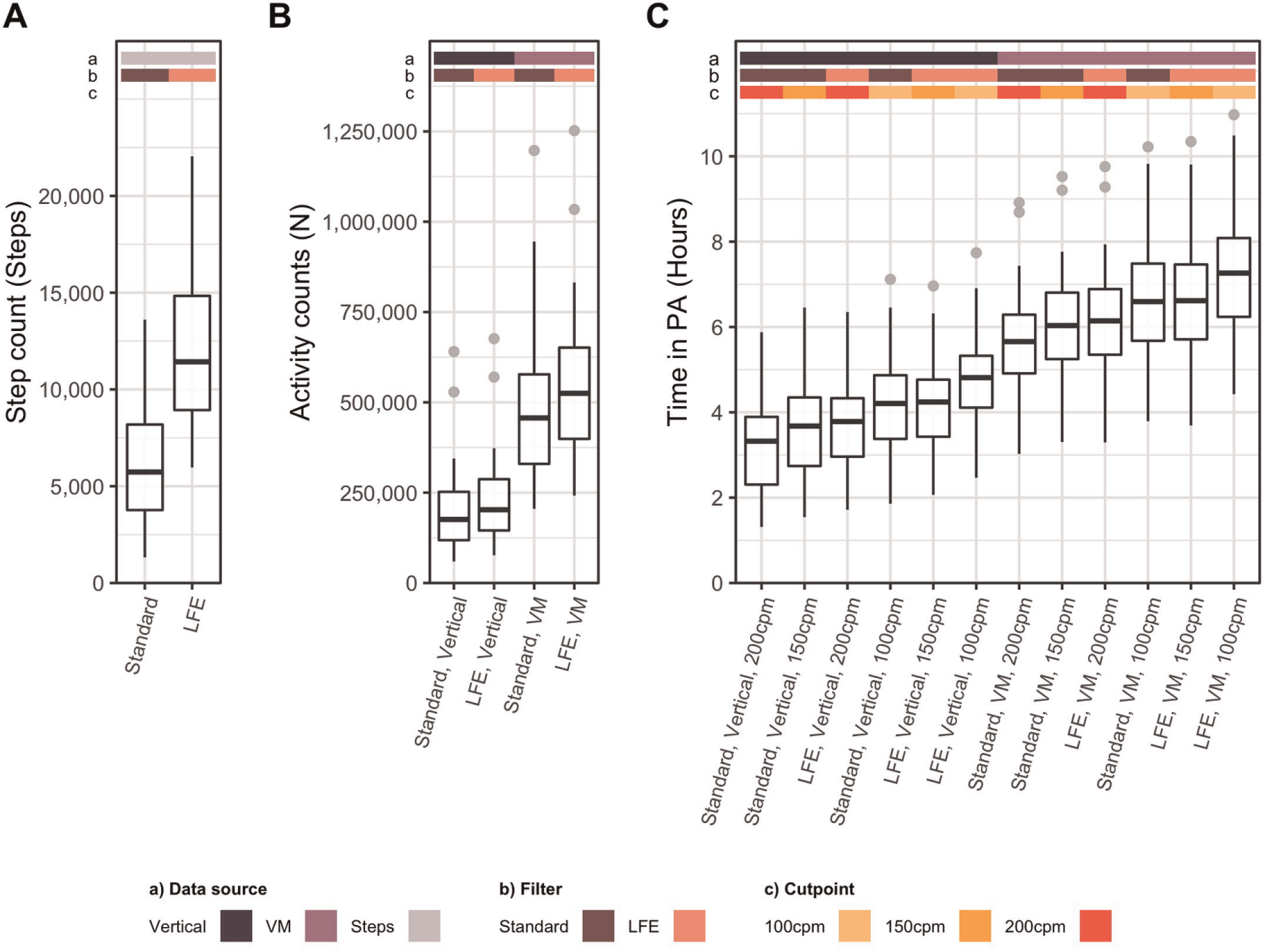
Physical activity metrics derived from the Actigraph GT3X. (**A**) Average daily step count derived through 2 methods, (**B**) Average daily activity counts derived through 4 methods, (**C**) Average daily time in PA derived through 12 methods. Methodological factors which affected each metric are noted as colored bars and labels on the x axis, including the data source [vertical vs. vector magnitude (VM) counts], filter [standard vs. low frequency extension (LFE)], and sedentary cut point [100 counts per minute (cpm), 150 cpm, 200 cpm].

Step counts derived from the two filtering methods differed (*p* < 0.001), with population medians of 5,852 and 11,695 steps per day when the Standard filter and LFE were used, respectively (Figure 2A). The correlation (Pearson's r) and agreement (CCC) between methods were 0.89 and 0.31, respectively (Supplementary Table S4).

#### 3.2.4 Activity counts

Regression showed that preprocessing filter (Standard vs. LFE) and data source (Vertical axis vs. VM) independently affected activity counts, but wear time algorithm and inactivity period did not. Activity counts decreased with increasing MS severity. Fully adjusted models identified interactions between filter and data source and effect modification due to disease severity, but these terms were not retained in reduced models. When adjusted for wear time, activity counts became independently affected by wear time algorithm and inactivity period. The Troiano algorithm and a 30 min inactivity period imparted the largest biases (Supplementary Table S3).

Unadjusted activity counts derived from the resulting four methods differed (*p* < 0.001) with population medians ranging from 179,324 to 533,753 counts per day (Figure 2B). In pairwise analysis (*n* = 6 pairs), all methods differed from one another (each *p* < 0.001) (Supplementary Table S4). Correlations between pairs ranged from 0.90 to 1.00. Agreement was high when methods used the same data source (CCC: 0.94–0.97) and very weak when data source differed (0.24–0.37). All pairwise differences, correlations, and agreement, including confidence intervals, are reported in Supplementary Table S4.

#### 3.2.5 Total time in physical activity

Preprocessing filter, data source, and sedentary cutpoint independently affected estimates of time in PA. The Troiano wear time algorithm and 30 min inactivity period yielded statistically significant, but negligible increases in time in PA. Regardless of method, time in PA decreased with increasing MS severity. The LFE increased estimates of time in PA compared to the standard filter, while increasing the sedentary cutpoint reduced time in PA. Though the use of VM counts increased time in PA for all levels of MS severity, it disproportionately increased time in PA for participants with severe MS. When time in PA was adjusted for wear time, it became dependent on wear time algorithm and inactivity period. In this analysis, the inactivity period term also exhibited interactions with filter, data source, and wear time algorithm terms. These interactions were mostly attributed to the sensitivity 30 min inactivity period to other methods (Supplementary Table S3).

Unadjusted total time in PA derived from the 12 resulting methods differed (*p* < 0.001), with population medians ranging from 3.3 to 7.5 h of PA per day (Figure 2C). In pairwise analysis (*n* = 66 pairs), all pairs except two differed significantly from one another (each *p* < 0.001) (Supplementary Table S4). In these two cases, differences caused by the filter and sedentary cutpoint offset each other. Correlations between methods ranged from 0.61 to 1.00, while agreement ranged from 0.10 to 1.00. Agreement tended to be highest for pairs which (1) used the same data sources and (2) used a higher cutpoint with the LFE compared to the standard filter (Supplementary Table S4).

#### 3.2.6 Sedentary behavior

Unlike other PA metrics, regression showed that the preprocessing filter, data source, cutpoint, wear time algorithm, and inactivity period definition all independently affected sedentary time estimates. Data source, wear time algorithm, and inactivity period exhibited interactions (i.e., effect modification) by disease severity. The effects of the filters exhibited interactions with wear time algorithm and inactivity period, and inactivity period and wear time algorithm exhibited interactions with each other. Adjusting for wear time reduced, but did not eliminate, the dependency of sedentary time on wear time algorithm and inactivity period (Supplementary Table S3).

**Figure 3:**
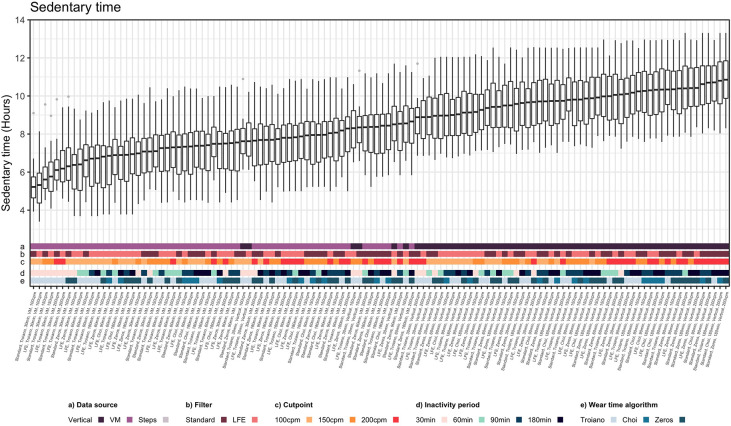
Sedentary time derived from the actigraph GT3X. Sedentary time estimates derived though 120 methods. Methodological factors which affected estimates of sedentary time are noted as colored bars and labels on the x axis, including the data source [vertical vs. vector magnitude (VM) counts], filter [standard vs. low frequency extension (LFE)], sedentary cutpoint [100 counts per minute (cpm), 150 cpm, 200 cpm], inactivity period (30, 60, 90, or 180 min), and wear time algorithm (Zeros, Troiano, or Choi).

Unadjusted sedentary time estimates stemming from the 120 possible combinations of methods significantly differed from each other (*p* < 0.001), ranging from 5.2 to 10.9 h of sedentary time per day (Figure 3). In pairwise analysis, 6,718 of the 7,140 pairs significantly differed from each other (Supplementary Table S4). Correlations between methods ranged from 0.15 to 1.00, whereas agreement (CCC) ranged from 0.03 to 0.99 (Supplementary Table S4).

#### 3.2.7 Time in light physical activity

Time spent in LPA was independently affected by sedentary cutpoint and MVPA cutpoint. The Troiano wear time algorithm and 30 min inactivity period yielded statistically significant, but negligible increases in time in LPA. Estimates of LPA decreased with increasing sedentary cutpoint. Vertical axis-based MVPA cutpoints which used LFE (Sandroff 1,722, Sandroff 1,745, Sandroff Severity, Freedson) registered more time in LPA than the Sandroff-1,584 cutpoint, which uses the standard filter. The Sasaki cutpoint, which is based on VM counts, yielded the highest time LPA. Time in LPA decreased in moderate and severe MS compared to mild MS, though MS severity was an effect modifier for some MVPA cutpoints (Sandroff 1,584, Sasaki, Sandroff Severity). In addition to these dependencies, adjustment for wear time made time in LPA dependent on wear time algorithm and inactivity period. These terms exhibited interactions both with each other, and multiple other methodological factors (Supplementary Table S3).

**Figure 4:**
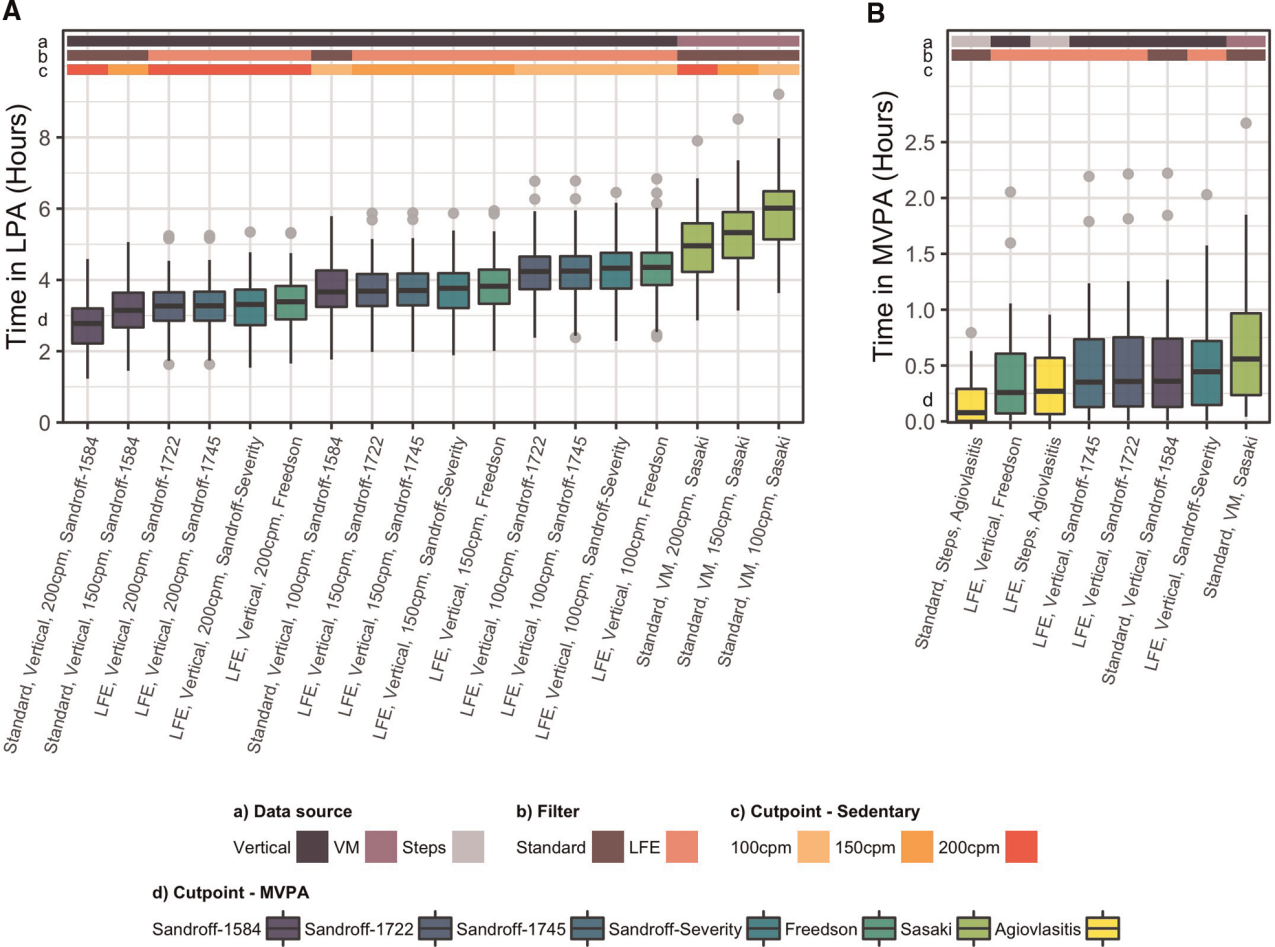
Time in light and moderate to vigorous physical activity derived from the actigraph GT3X. (**A**) Average time in light physical activity (LPA) derived through 18 methods, and (**B**) Average daily time in moderate to vigorous physical activity (MVPA) derived through eight methods. Methodological factors which affect each metric are noted as colored bars and labels on the x axis, including the data source [vertical vs. vector magnitude (VM) counts], filter [standard vs. low frequency extension (LFE)], sedentary cut point [100 counts per minute (cpm), 150 cpm, 200 cpm], and MVPA cutpoint.

The 18 methods of calculating time in LPA significantly differed from each other (*p* < 0.001), with population medians ranging from 2.8 to 6.1 h per day (Figure 4A). In pairwise analysis, 141 of the 153 resulting pairs significantly differed from each other (*p* < 0.001) (Supplementary Table S4). Correlations between methods ranged from 0.61 to 1.00 and agreement ranged from 0.09 to 1.00. The Sasaki method, which was the only VM-based method, consistently exhibited the lowest correlations and agreement with all other vertical axis based methods.

#### 3.2.8 Time in moderate to vigorous physical activity

Cutpoint, but not wear time algorithm or inactivity period definition, independently affected estimates of time in MVPA. Vertical axis based methods which were developed for MS populations (Sandroff 1,584, Sandroff 1,722, Sandroff 1,745, Sandroff Severity) did not differ from each other at the population level. The Freedson method, which was developed for healthy adults, and the Agiovlasitis method, which was based on step cadence, registered less time in MVPA than these methods. The Sasaki method, which was based on VM counts, yielded the highest time in MVPA. Time in MVPA decreased in moderate and severe MS compared to mild MS, but MS severity was not an effect modifier. Adjustment for wear time caused statistically significant, though negligible increases to time in MVPA (Supplementary Table S3).

The eight methods of calculating time in MVPA significantly differed from each other (*p* < 0.001), ranging from population medians of 5 to 36 min per day (Figure 4B). Twenty-two of the 27 pairs significantly differed from each other in pairwise analysis (Supplementary Table S4). The four vertical axis based methods (Sandroff-1584, Sandroff-1722, Sandroff-1745, Freedson) demonstrated almost perfect correlation and agreement with each other (*r*: 0.99–1.00, CCC: 0.96–1.00), and pairings of these methods and the Sasaki method (VM-based) or the Sandroff-Severity method (applied different cutpoints across MS severity strata) also exhibited high correlation and agreement (*r*: 0.79–0.91; CCC: 0.72–0.88). The Agiovlasitis method, which was based on step cadence, consistently exhibited the lowest correlation and agreement with other methods (*r*: 0.59–0.79, CCC: 0.23–0.52) (Supplementary Table S4).

#### 3.2.9 How can methodological heterogeneity bias PA research?

##### 3.2.9.1 Meeting PA recommendations

Using this study as an illustrative example, 92.9% of participants would have met a 7,500 step target using the LFE, whereas only 33.3% would have met this threshold with the standard filter (Table 6). Similarly, the percentage of the population which met an MVPA target of 30 min per day ranged from 14.3% with the Agiovlasitis method with the standard filter to 59.5% using the Sasaki method (Table 6).

**Table 6:** Percentage of study participants meeting physical activity guidelines according to multiple methods.

Method	% meeting PA guidelines
7,500 steps per day	
Standard filter	33.3%
LFE	92.9%
30 min of MVPA per day	
Sandroff 1584	40.5%
Sandroff 1722	40.5%
Sandroff 1745	38.1%
Freedson	31.0%
Sasaki	59.5%
Agiovlasitis (Standard filter)	14.3%
Agiovlasitis (LFE)	26.2%

##### 3.2.9.2 Relationships between PA and clinical outcomes

Correlations with clinical measures varied across methods for all PA metrics (Supplementary Table S5). Both step count methods exhibited moderate to strong correlations with the EDSS, 6 MWT, 10 mWT, and MSWS-12, though point estimates tended to be higher for the standard filter than for the LFE. For all other metrics, vertical axis based methods tended to exhibit stronger correlations with clinical measures than VM based methods. Time in LPA did not consistently correlate with clinical measures *via* any method. No objectively-measured PA metric correlated with the IPAQ. Adjustment for wear time did not consistently affect the associations between PA metrics and clinical outcome measures.

## 4 Discussion

In this rapid review and comparative analysis, we found that existing literature on PA in MS populations exhibited high methodological heterogeneity, and methods were often poorly reported. Through direct comparison, we found that all PA metrics were sensitive to methodological heterogeneity, though the degree and uniformity of this sensitivity varied. Heterogeneity affected the outcome of analyses relevant to the MS literature, demonstrating potential confounding effects on population-level findings and meta-analyses. Though this study was not designed to assess the relative accuracy or validity of included methods and metrics, our results suggest several PA metrics and methods which should be used and interpreted with caution in MS populations. We therefore make the following recommendations to guide future research with the Actigraph GT3X:

• Improved reporting and adherence to validated methods are needed
• Vertical axis counts may be preferable to VM counts for all PA metrics
• Step counts derived from hip-worn Actigraph GT3X are not recommended
• Time in PA may be preferable to sedentary time as an indicator of sedentary behavior
• Sedentary cutpoints require further research to accurately characterize light PA
• MVPA derived from different data sources are not comparable
• The Troiano algorithm and 30 min inactivity periods are not recommended

### 4.1 Improved reporting and adherence to validated methods are needed

Our comparative analysis demonstrated that methodological factors have the potential to confound common PA metrics. However, we also showed that the reporting of Actigraph data processing methods is both limited and inconsistent in existing literature. These findings demonstrate that our ability to interpret and compare studies is limited, and highlight the need for improved methodological reporting in studies which use the Actigraph GT3X. At a minimum, authors should report the data source, filter, wear time algorithm, cutpoints, and sensor placement they used to derive their PA metrics. We also identified several instances in which established methods were altered when deriving PA metrics (eg, using the LFE when the method was developed for use with the standard filter). We have shown that these alterations may affect the validity of the resulting PA metrics, and recommend that authors adhere to the filters, sensor placements, and data sources for which specific cutpoints were developed. Finally, given the relatively large impact of methodological factors on study outcomes, data processing methods should also be specified a-priori.

### 4.2 Vertical axis counts may be preferable to VM counts for all PA metrics

Of the methodological factors assessed here, discrepant data sources consistently yielded the largest differences in PA estimates. PA metrics derived from vertical axis and VM counts exhibited low agreement and correlation with each other, with VM counts yielding significantly higher PA estimates than vertical axis based methods. Metrics derived from these data sources behaved differently across disease severity strata, with VM based metrics disproportionately increasing PA estimates in those with severe MS. As a result, vertical axis based methods consistently exhibited stronger correlations with clinical measures, suggesting superior construct validity. We therefore do not recommend direct comparisons between studies which derive PA from different data sources, and recommend the use of vertical axis based methods over VM based methods for all PA metrics evaluated here.

### 4.3 Step counts derived from hip-worn Actigraph GT3X are not recommended

Daily step count was one of the most common PA metrics studied in our review, though methods used to derive step count were not commonly reported. In our review, only six of the 26 studies which measured step count reported whether they used the standard filter or the LFE. However, we showed that filter selection strongly affected step count estimates, with the LFE nearly doubling step counts relative to the standard filter. This effect was inconsistent across disease severity strata, disproportionately increasing PA estimates in those with severe MS.

The manufacturer of the Actigraph recommends using the LFE in populations which move slowly or take very light steps, though they do not define a specific threshold below which the LFE should be used (16). In an independent study, Bezuidenhout et al. showed that the standard filter and LFE perform similarly when walking speeds exceed 1.0 m/s during scripted, over-ground walking tasks, whereas the LFE is more accurate at walking speeds below 1.0 m/s (18). However, Feito et al. showed that the standard filter underestimates step count by 25%–30% and the LFE overestimates step count by 30% when daily step counts are evaluated under free-living conditions (108). Feito et al. hypothesized that these biases were due to reduced sensitivity to slow walking and mischaracterization of non-walking movement, respectively (108).

In this study, 16 of the 42 participants (38%) exhibited normal walking speeds slower than 1.0 m/s, suggesting that the LFE may be preferable in our population based on Bezuidenhout et al.'s findings (18). However, like Feito et al., we found that step counts derived with the LFE were unrealistically high for those of all MS severity levels (108). In addition, we found that step counts derived using the standard filter exhibited stronger correlations with clinical measures. However, due to the standard filters’ propensity to underestimate step count in slow walkers, it is probable that these relationships are confounded by the relationship between MS severity and walking speed. For these reasons, we caution researchers against using an Actigraph GT3X worn on the hip to measure step counts. If step counts are desired as PA outcomes, other devices or ankle placement of the Actigraph have been shown to provide higher accuracy (18, 108, 109).

### 4.4 Time in PA may be preferable to sedentary time as an indicator of sedentary behavior

Sedentary behavior is a relevant outcome in MS populations (110) and was the second most frequently studied PA metric in our review. However, we found that sedentary time estimates are highly sensitive to data processing methodology and wear time definitions, consistent with findings in a healthy population (111). Adjustment for daily wear time has been used to account for the relationship between sedentary time and wear time, reducing inter-personal variability arising from differences in wear time (21, 38–40). We found that such adjustment reduced, but did not eliminate, the biases imparted by sedentary time's dependency on wear time or other methodological factors. In addition, the methods affecting sedentary time estimates exhibited interactions and were poorly reported in the literature. This reporting gap and the multiple interactions exhibited between methodological factors in our models make it nearly impossible to account for independent effects of individual factors or control for differences across studies.

We found that total time in PA, which is effectively the inverse of sedentary time, is not sensitive to wear time definition and exhibits fewer interactions between methodological factors. Though our rapid review showed that time in PA is not widely used as an outcome in the MS literature, it may nevertheless be a preferable, albeit indirect, indicator of sedentary behavior until the relative validity of sedentary time methods is clarified. In addition, we recommend using vertical axis counts rather than VM counts to derive time in PA, as this method consistently exhibited stronger relationships with clinical measures.

### 4.5 Sedentary cutpoints require further research to accurately characterize light PA

Kozy Keadle et al. previously assessed the validity of multiple sedentary cutpoints in overweight adults using vertical axis counts and the LFE, recommending 150 cpm in that population with those sensor settings (112). In a healthy population, Carr and Mahar found that 100 cpm is an appropriate cutpoint for single-axis measurements with the Actigraph GT1M (113), which is roughly equivalent to the vertical axis based measures using the LFE on the GT3X (25). They also found that a cutpoint of 150cpm provides higher accuracy in VM based measurements without the LFE (113). However, both of these studies aimed to assess accuracy with respect to sedentary behavior, and both cutpoints exhibited lower accuracy when identifying light physical activity. We cannot recommend one cutpoint over the others based on this study. However, given our recommendation to use time in PA rather than sedentary time, we do recommend further study of sedentary cutpoints to identify thresholds which accurately identify time in light physical activity. During this research, cutpoints should be confirmed for both the standard filter and the LFE. We also suggest that researchers more readily report their cutpoints, data sources, and filters to enable comparisons across studies.

### 4.6 MVPA derived from vertical axis, VM, and step counts are not comparable

Time in MVPA was the most frequently-used PA metric identified by our rapid review. Though eight methods were used to derive MVPA, four methods derived from vertical magnitude counts performed similarly (Sandroff-1584, Sandroff-1722, Sandroff-1754, and Freedson). Three of these methods – Sandroff-1584, Sandroff-1722, and Sandroff-1754 – were essentially equivalent and exhibited strong, consistent correlations with clinical measures. The Freedson method employed a higher cutpoint originally developed for a healthy population, and therefore yielded lower estimations of PA (104). However, it yielded nearly identical correlations with clinical measures as the other vertical axis based methods. Together, these four methods were used in the majority of records identified in our review.

The Sandroff-Severity method applies different cutpoints to data from individuals with mild, moderate, and severe MS, as individuals with severe MS exert more energy than those with mild and moderate MS to complete the same tasks, thereby lowering the threshold at which activities qualify as “moderate to vigorous” PA (24, 33, 106). It is therefore unsurprising that this method demonstrated lower agreement and lower correlations with clinical measures compared to other methods. Sandroff et al. derived these cutpoints for sub-populations in MS, but did not necessarily intend for them to be used as a single method (24). It is currently unclear which of these approaches – uniform cutpoints or severity-specific cutpoints – yield more valid population-level and individual-level representations of MVPA. Direct comparisons with a gold standard may be beneficial in future research.

The Agiovlasitis method, which is based on step cadence, yielded the lowest estimations of MVPA and exhibited the lowest correlation and agreement with other methods. MVPA estimates made though this method were highly sensitive to choice of filter, since they were derived from step counts. However, both implementations of this method yielded similar correlations with clinical measures as the vertical axis based measures. This method is therefore not directly comparable to vertical axis based methods, but nevertheless demonstrates evidence of construct validity.

The Sasaki method, which is based on VM counts, exhibited only moderate correlations with clinical measures, moderate correlations with other methods, and yielded by far the highest estimate of MVPA time. Similar to other PA metrics derived from VM counts, we cannot recommend the use of the Sasaki method until additional research establishes its validity.

### 4.7 The Troiano algorithm and 30 min inactivity periods are not recommended

When PA metrics were affected by wear time methods, they were consistently the most strongly biased by the Troiano algorithm and the use of a 30 min inactivity period. These methodological options enable shorter inactivity bouts, with or without activity spikes, to be counted as non-wear time. While this bias only affected sedentary time estimates in unadjusted PA metrics, it affected all PA metrics which were adjusted for wear time. The Troiano algorithm continues to be widely used in the physical activity literature (15), though it has been found to substantially underestimate wear time when it is used with hip-worn Actigraphs (107). This is consistent with our own findings, as the wear time estimates produced by the Troiano algorithm were substantially lower than those produced by the Choi algorithm or continuous zeros. The remaining two algorithms – Choi algorithm and continuous zeros – were not equivalent. However, they imparted smaller relative biases on both unadjusted and adjusted PA metrics compared to the Troiano algorithm. For these reasons, we recommend that researchers adopt either the Choi algorithm or the continuous zeros method to calculate wear time. We further recommend that wear time is calculated with an inactivity period of at least 60 min due to the level of bias induced and the number of interactions exhibited by the 30 min wear period.

### 4.8 Future research

These findings highlight that further research is needed to establish both the absolute and relative validity, reliability, responsiveness of PA metrics derived through different data processing methods. This research should include not only clinical assessments of scripted PA, but also validation compared to a reference standard during free-living assessments (114), as many of the methods studied here were developed and validated only during scripted walking tasks in clinical settings (23, 32, 69, 104). Once the relative validity, reliability, and responsiveness of these methods are established, an optimal set of PA metrics derived from the Actigraph GT3X can be defined for MS populations.

### 4.9 Strengths and limitations

This study reviews existing literature, quantifies methodological heterogeneity associated with the most widely-used PA monitor in MS, and illustrates the potential consequences of this heterogeneity on PA metrics through direct, pairwise comparisons. We offer a comprehensive exploration of the impact of methodological heterogeneity on Actigraph-based PA metrics in MS populations.

However, this study is not without its limitations. The summary of the existing literature is based on a rapid review rather than a systematic review, and therefore made compromises to ensure feasibility. However, the methods identified in the review appeared to reach saturation, defined as the point at which new data are unlikely to alter conclusions, and it is unlikely that we missed any commonly-used data processing methods. For PA metrics which rely on cutpoints, such as LPA and MVPA, we found that studies often deviated from the filters and data sources for which specific cutpoints were developed. We did not assess the effects of every possible combination of filter, data source, and cutpoint on these metrics. However, deviations from established methods are likely to further increase variability in PA literature. In our analysis, PA metrics were derived through various methods from the same raw data, introducing dependencies which could introduce over-fitting and bias standard errors or *p* values. However, we mitigated this risk by adjusting for participant-level random effects, using BIC values (rather than *p* values or AIC) to guide model development, using paired analysis methods where appropriate, and adjusting analyses for multiple testing. Quantitative comparisons were conducted based on data collected during and immediately after inpatient rehabilitation, which included structured exercise training and physiotherapy. The PA behavior described here is therefore not necessarily representative of free-living PA. Finally, PA monitors other than the Actigraph GT3X are not considered here, and care should be taken when comparing findings between devices.

## 5 Conclusions

Though the Actigraph GT3X is widely used and is often considered well-validated in MS populations, the PA metrics derived from this device are sensitive to methodological choices during data processing. Methodological heterogeneity of existing literature is high, and additional research is needed to establish the relative validity and responsiveness of existing methods. Researchers should consider the impact of methodological heterogeneity on PA metrics when selecting and reporting their methods, as these decisions may limit the responsiveness, interpretability, and cross-study comparability of PA outcomes.

## Data Availability

The raw data supporting the conclusions of this article will be made available by the authors, without undue reservation.
